# Our Journal

**Published:** 1859-01

**Authors:** 


### OUR JOURNAL.

From various circumstances beyond control, the January number of the Journal has been delayed, greatly beyond the usual time of issue. We wish it distinctly understood that this is no evidence of a want of usual interest in its welfare upon the part of its Editors; so far from it, they regard the present as rather the dawn of a still brighter future, and so far from relaxing their energies, are determined to renew their exertions in its behalf, with the opening of another year.

This they have encouragent to do, from the evidences of appreciation of their labors, received from so many sources in the last few months, in the strong tide of payments which have been setti g in upon the Journal. We desire to say, however, to our friends, that it is not alone from money, that a Journal can be sustained. Send on your contributions for its pages.

